# Effect of hyperbaric oxygenation on random rat skin flaps vascularization

**DOI:** 10.1590/ACB360906

**Published:** 2021-11-08

**Authors:** Fabricio Valandro Rech, Ricardo Santos Simões, Jefferson André Pires, Rinaldo Florêncio-Silva, Djalma José Fagundes

**Affiliations:** 1 Fellow PhD degree. Postgraduate Program in Interdisciplinary Surgical Sciences - Universidade Federal de São Paulo (UNIFESP) – Sao Paulo (SP), Brazil.; 2 PhD. Department of Obstetrics and Gynecology - Universidade Federal de São Paulo (UNIFESP) – Sao Paulo (SP), Brazil.; 3 Fellow PhD degree. Postgraduate Program in Biophotonics Applied to Health Sciences - Universidade Nove de Julho (UNINOVE) - Sao Paulo (SP), Brazil.; 4 PhD. Department of Gynecology - Universidade Federal de São Paulo (UNIFESP) – Sao Paulo (SP), Brazil.; 5 PhD, Full Professor. Division of Surgical Techniques and Experimental Surgery - Department of Surgery - Universidade Federal de São Paulo (UNIFESP) – Sao Paulo (SP), Brazil.

**Keywords:** Surgical Flaps, Hyperbaric Oxygen, Vascular Endothelial Growth, Rats

## Abstract

**Purpose:**

To evaluate the effect of hyperbaric oxygenation (HBO) on angiogenesis in random rat skin flaps, by immunoexpression of vascular endothelial growth factor A (VEGF-A).

**Methods:**

Forty adult rats were divided into four groups: GE) epilated; GE/HBO) epilated subjected to HBO; GER) epilated submitted to dorsal skin flap; GER/HBO) epilated subjected to dorsal skin flap + HBO. HBO was performed with rats inside a chamber under atmosphere close to 100% oxygen and pressure of 2.4 absolute atmospheres, 2h per day during seven consecutive days. GE and GER groups were placed in the hyperbaric chamber without HBO. Then, under anesthesia, skin flaps were removed and separated into three portions relative to pedicle fixation. The samples were fixed in formalin and processed for paraffin embedding. Histological sections were submitted to immunohistochemistry for VEGF-A detection. The number of immunostained-blood vessels were counted under light microscopy.

**Results:**

GE and GE/HBO groups showed normal and similar skin morphology in the three flap portions. A fibrin-leukocyte crust, along with denatured collagen and intense leukocyte infiltrate, was mainly observed in the dermis of the medial and distal flap portions of GER group. Meanwhile, the GER/HBO group presented more regions with intact collagen and small areas of leukocyte infiltrate in the three flap regions. VEGF-A-immunostained blood vessels were largely seen in all regions of GE and GE/HBO groups, whereas no significant differences were found between these groups. A decrease in vascularization was noticed in GER and GER/HBO groups, which was more evident in the most distal portion of the flaps. However, the number of VEGF-A-immunostained blood vessels in GER/HBO group was significantly higher when compared to GER group.

**Conclusions:**

Hyperbaric oxygenation was associated with increased angiogenesis and improved viability of rat skin flaps.

## Introduction

Injuries to the integumentary system with loss of substance, from different causes, can be treated with the use of skin flaps. Surgical treatment, despite planning and execution with careful techniques, can result in reconstructive failure. Optimizing the feasibility of surgical flaps has always been a challenge in medical science. A viable flap implies a decrease in post-surgical morbidity, a faster recovery of patients and a lower cost to the healthcare system involved^1^^-^ 7.

McFarlane *et al*.^8^ standardized an experimental model in rats to assess the viability of random flaps. They concluded that biochemical and morphological changes take place according to the distance from the tip of the flap to its bottom (pedicle). The ischemia to which the flap is submitted, especially in the first hours of its application, is essential to determine the extent of its viability. The presence of hematoma and edema, and the relation of the pedicle width versus skin flap area itself can lead to necrosis of the distal portion of the flap to the pedicle in percentages ranging from 9 to 65%, according to the experimental model tested^9^^-^ 11.

Hyperbaric oxygen therapy (HBO) has been mainly used as an adjuvant therapeutic resource in several diseases, with emphasis on the response of injured tissues in healing processes with less surgical reinterventions and lower morbidity. Moreover, HBO is commonly used as treatment in several diseases, such as non-healing chronic wounds^12^. Conceptually, it is the intermittent therapeutic administration of oxygen in a chamber with an atmospheric pressure greater than that observed at sea level (1 atmosphere). Thus, HBO is based on the dilution of oxygen in body fluids, which are usually under a pressure between 2 and 3 atmospheres absolute (ATA). For safety purposes, the hyperbaric chamber must withstand up to 1.5 times the maximum working pressure (4.5 ATA)^1^^,^ 2 ^,^ 4 ^,^ 6.

HBO has been proposed for the treatment of several models of compromised flaps and, when instituted immediately after surgery, it can mitigate the progression of ischemia, which leads to apoptosis and necrosis^1^^,^ 3 ^,^ 13. There is experimental evidence regarding hyperbaric oxygenation in various types of tissues and organs^14^^-^ 23. The administration of oxygen under pressure causes tissue hyperoxygenation, vasoconstriction, fibroblast activation, modulation of the inflammatory response, synthesis of growth factors, antibacterial effects, and reduction of leukocyte chemotaxis, factors that are related to favor the healing process^7^^,^ 17 ^,^ 24.

Based on these previously related benefits of HBO, other studies are needed to best understand the mechanisms of HBO in the tissue vascularization process. Thus, the aim of this article was to evaluate the effect of hyperbaric oxygenation on angiogenesis in dorsal skin flaps of rats, by immunohistochemical expression of vascular endothelial growth factor A (VEGF-A).

## Methods

The experimental protocol of this study was approved by the Ethics Committee of the Universidade Federal de São Paulo (UNIFESP-CEUA), under the number 431,182. The experimental procedures followed the international standards for animal research and the normative guidelines of the Brazilian Society for Science in Laboratory Animals (SBCAL).

Forty adult rats (*Rattus norvegicus albinus*), with body weight between 280 and 320 g, were kept in the laboratory facility of the Department of Universidade Regional Integrada do Alto Uruguai e das Missões (URI), in Erechim (RS, Brazil). The animals were housed in individual cages and maintained in an environmentally controlled laboratory (12-h light/dark cycle at 25°C), with access to food and water *ad libitum*.

After an adaptation period, the animals were randomized into four groups (n = 10 each):

- GE: animals that underwent epilation;
- GE/HBO: animals that underwent epilation and were subjected to HBO;
- GER: animals that underwent epilation and were subjected to dorsal skin flaps;
- GER/HBO: animals that underwent epilation and were subjected to dorsal skin flaps and HBO.

### Anesthetic procedure and analgesia

After 6 and 4 h of fasting for solid and liquid diets, respectively, the animals received intramuscular injection of acepromazine (acepromazine maleate, 5 mg/kg, C-Vet Veterinary Products, United Kingdom). After 10 min, the rats received a combined intramuscular injection of ketamine hydrochloride (50 mg/kg, Vetalar, Parke-Davis, United Kingdom) and xylazine hydrochloride (10 mg/kg, Xilazin, Syntec, Brazil). The animals received aspirin (100 mg/Kg) diluted in water until the day of euthanasia, without antimicrobial prophylaxis.

### Surgical procedures

After anesthesia, the animals were epilated on dorsal area under antiseptic condition (0.2% chlorhexidine gluconate, Riohex, Biofarma, Brazil, as antiseptic solution). A rectangular cutaneous area comprising 2 cm in width and 8 cm in length (proportion 1 × 4) was bounded with a permanent pen marker.

**Figure 1:**
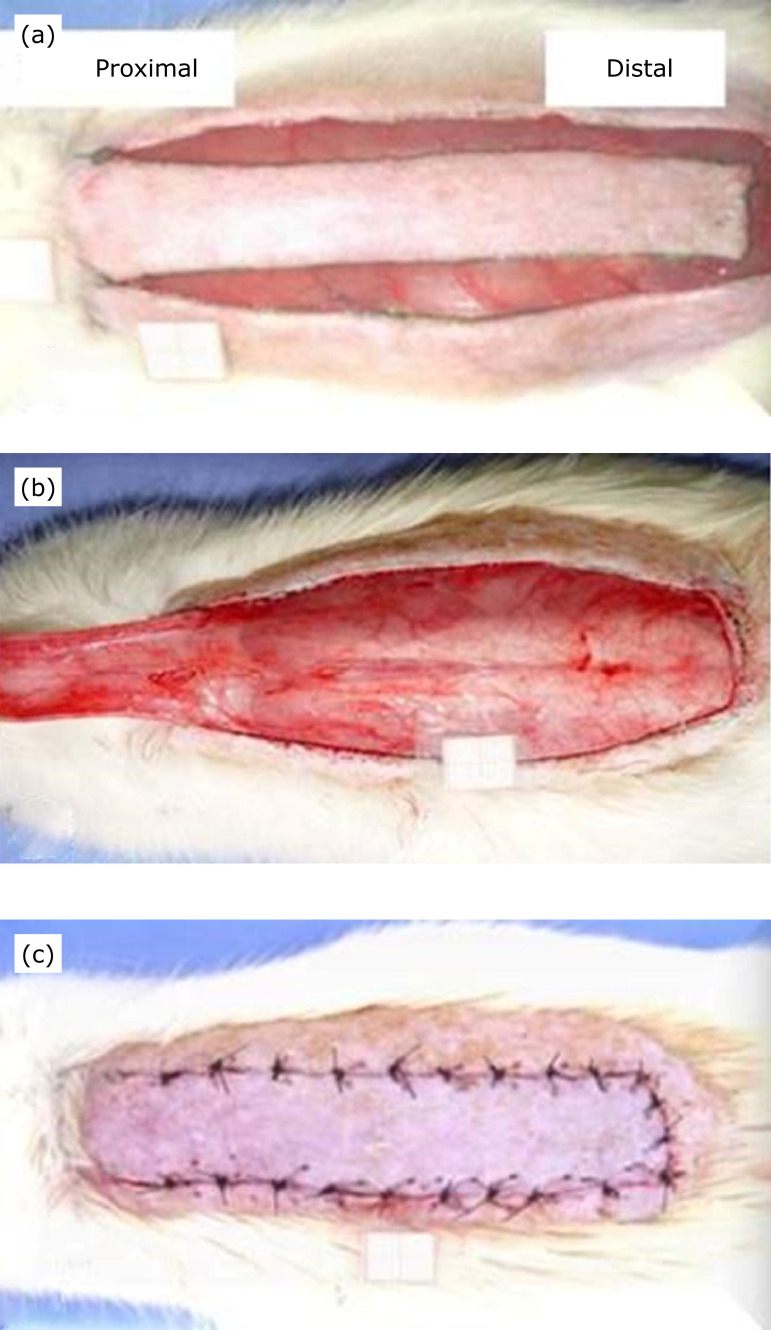
**(a)** Skin incision of distal and lateral edges before flap detachment, **(b)** detail of the raised flap, and **(c)** final aspect of interrupted suture of skin flap.

At the limits of the delimited skin area, an incision was made on the three edges of the delimited area with a scalpel blade (no. 15), maintaining the continuity of the flap along its cranial portion, according to the model proposed by McFarlane and modified by our group^1^. The flap was composed of epidermis, dermis, adipose tissue, and *panniculus carnosus* (skeletal muscle), which raised and was maintained fixed only by its pedicle (cephalic portion of the rectangle). The flap was repositioned in its original place and sutured with interrupted suture of 4-0 nylon monofilament (Fig. 1 a-c).

### Hyperbaric chamber

The animals were placed in the hyperbaric chamber in accordance with their group. HBO was carried out in an appropriate chamber for animal experimentation developed by the team involved in this research^5^. Prior to pressurization, oxygen injected into the chamber for 5 min displaced the environmental air inside it and allowed for an atmosphere close to 100% oxygen. The chamber’s internal pressure increased at constant rate, progressively, until reaching the pressure of 2.4 ATA.

### Procedure of hyperbaric oxygenation

HBO was daily performed in GE/HBO and GER/HBO groups. Ten animals, in individual stalls for each section, stayed under continuous oxygen flow. Each daily section lasted 2 h during seven consecutive days, always performed at the same time. The animals of GE and GER groups were placed in the hyperbaric chamber, but they were not subjected to HBO procedure.

### Postoperative follow up

The clinical conditions of all animals were daily observed during the postoperative period. The wounds were daily examined to identify signs of inflammation, discharge, dehiscence, or infection.

### Procedure of incisional biopsies

On the eighth postoperative day, the animals were anesthetized by ketamine (50 mg/kg) and xylazine (10 mg/kg).After carefully positioning the rats in the operating board, incisional biopsies of 1 cm × 1 cm were collected at the proximal, middle, and distal area of the flap. Then, the areas were defined as follows:

- Area A: proximal;
- Area B: medial, located between the proximal and distal areas;
- Area C: distal to the pedicle implantation;
- Area D: fragment withdrawn outside flap located at 2 cm from the suture line (control fragment).

The samples were immediately immersed in fixative solution and submitted to standard histological processing.

### Euthanasia

After incisional biopsies with the animals still under anesthesia, the animals were euthanized by anesthetic depth. All the biological material was discarded in accordance with the current facilities rules of the Ethics Committee on Animal Use (CEUA) of URI.

### Histological and immunohistochemical analysis

Skin samples were fixed in 10% formaldehyde for 12 h and then processed for paraffin embedding. Sections (5-µm thick) collected onto silanized slides were dewaxed in xylene, hydrated in decreasing concentrations of ethanol, and submitted to immunohistochemical reactions for the detection of VEGF-A. For this purpose, endogenous peroxidase activity was blocked by incubating the sections with 3% hydrogen peroxide for 5 min. The sections were incubated in a sodium citrate buffer (pH=6), 10 mM at 95°C for 20 min, and non-specific binding sites were blocked with 2% phosphate buffered saline (PBS)-bovine serum albumin (BSA) for 1 h. Sections were then incubated overnight in the primary antibody anti-VEGF-A (sc-57496, Santa Cruz Biotechnology, United States), diluted at 1:200. Afterwards, the sections were incubated in a biotinylated goat anti-mouse/rabbit (Ig, Duet kit Dako) secondary antibody, and reactions were revealed with the streptavidin-peroxidase system (DakoCytomation, United States) using 3,3’-diamino-benzidine (DAB) as a chromogen and counter-stained with hematoxylin. As a negative control, primary antibody was replaced by non-specific immunoglobulin (DAKOCytomation, United States).

Images of immunostained sections were captured with a high-resolution digital camera (AxioCam-MCR by Carl Zeiss) adapted to a light microscope (AxioLab, Carl Zeiss), with 40X objectives, for the evaluation of blood vessels immunostained to VEGF-A. A total of two sections (with at least 100-µm distance between sections) per animal were evaluated. Ten fields per section covering an area of 0.015 mm^2^ were analyzed, totaling 200 fields and 3 mm^2^ per group. In each field, the number of blood vessels immunopositive to VEGF-A was counted, and results were estimated as number/mm^2^.

### Statistical study

The statistical analyses were performed with GraphPad Prism 5.0^®^ software. Data were subjected to analysis of variance (ANOVA), complemented by the Tukey post-hoc test. Statistical significance was considered at p<0.05.

## Results

GE and GE/HBO groups had normal and similar skin morphology in the three portions of the flap. The three portions showed keratinized stratified squamous epithelium, along with dermis constituted by loose and dense connective tissue in the papillary and reticular portions, respectively. On the other hand, the groups submitted to skin flaps (GER and GER/HBO) presented keratinized stratified epithelium and papillary and reticular dermis rich in leukocytes, in the proximal portion. The medial and distal regions of GER group exhibited a fibrin-leukocyte crust and dermis with denatured collagen, as well as an intense infiltrate of leukocytes, which was more evident in the dermis. GER/HBO group showed keratinized stratified epithelium and dermis with integral collagen and denatured part infiltrated by leukocytes, in the proximal and medial flap regions. However, a small area with leukocyte crust and dermis showing intact and denatured collagen rich in leukocytes was noticed in the distal region of flaps.

**Figure 2:**
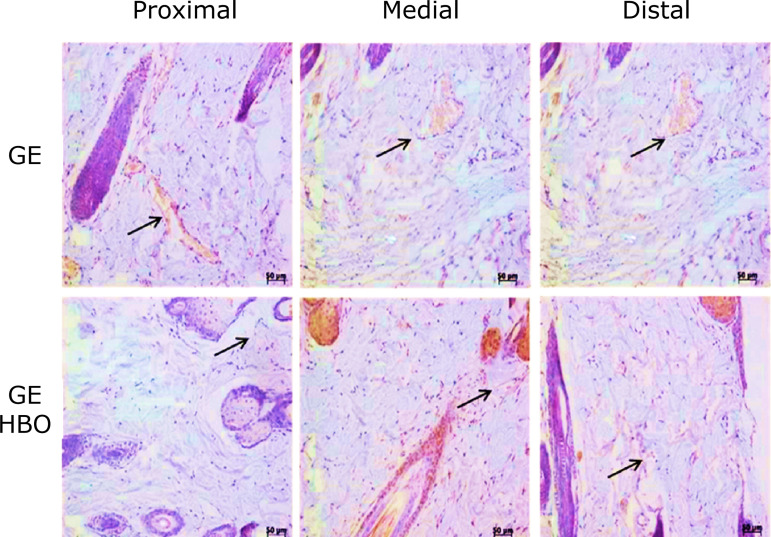
Photomicrographs of histological sections of skin fragments (proximal, medial, and distal regions) from GE and GE/HBO groups, subjected to immunohistochemistry for VEGF-A detection and counterstained with Harris’ hematoxylin. A similar pattern of blood vessels (*arrows*) immunopositive to VEGF-A were noticed in all analyzed regions. Magnification: x200.

GE: animals submitted to epilation; GE/HBO: animals submitted to epilation subjected to hyperbaric oxygenation; VEGF-A: vascular endothelial growth factor A.

Blood vessels immunopositive to VEGF-A were largely observed in all regions (proximal, medial, and distal) in the skin flaps of GE and GE/HBO groups, whereas a similar pattern in VEGF-A immunostaining were noticed in those groups. Indeed, the number of blood vessels immunopositive to VEGF-A, in the three regions studied, did not differ significantly between GE and GE/HBO groups (Fig. 2 and Table 1).

**Table 1:** Means and standard deviations of the number of blood vessels per mm^2^ in the four groups.

Region	GE	GE/HBO	GER	GER/ HBO
Proximal	262.10 ± 21.34 a	260.11 ± 31.23 a	134.30 ± 11.53 c	194.37 ± 25.36 b
Medial	260.14 ± 22.56 a	259.12 ± 51.24 a	45.20 ± 12.60 e	150.25 ± 11.34 c
Distal	252.35 ± 11.64 a	258.14 ± 11.55 a	22.30 ± 19,49 e	98.81 ± 11.90 d

HBO: hyperbaric oxygenation; GE: animals submitted to epilation, without skin flap and without HBO; GER: animals submitted to epilation, with a skin flap and without HBO; GE/HBO: animals submitted to epilation, without skin flap and with HBO; GER/HBO: animals submitted to epilation, with skin flap and with HBO;

a>b>c>d>e (p<0,05). Kruskal-Wallis test.

**Figure 3:**
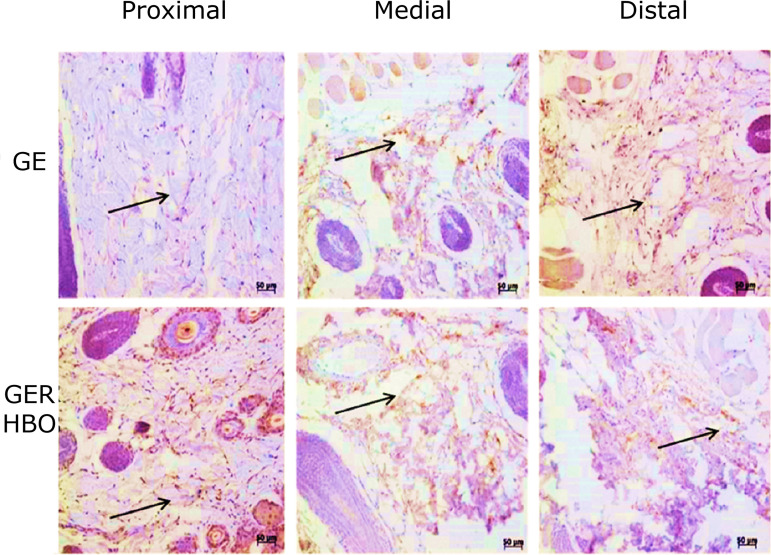
Photomicrographs of histological sections of skin fragments (proximal, medial, and distal regions) from GER and GER/HBO groups, subjected to immunohistochemistry for VEGF-A detection and counterstained with Harris’ hematoxylin. Blood vessels (*arrows*) immunopositive to VEGF-A were most noticed in the GER/HBO group. Magnification: x200.

GER: animals submitted to epilation submitted to dorsal skin flap; GER/HBO: animals submitted to epilation subjected to dorsal skin flap + hyperbaric oxygenation; VEGF-A: vascular endothelial growth factor A.

However, in the groups that underwent epilation with skin flaps without HBO (GER) and with HBO (GER/HBO), there was decrease in vascularization, which was more evident in the most distal portion of the flaps of both groups. It should be mentioned that the number of blood vessels immunopositive to VEGF-A was higher in all regions in the group with skin flaps submitted to hyperbaric oxygenation (GER/HBO), when compared to animals with skin flaps without HBO (Fig. 3 and Table 1).

## Discussion

Random skin flaps are frequently used to repair large areas of tissue loss. The technique is easy to perform and has low morbidity^25^. Nevertheless, even performed under the planning and execution of appropriate techniques, it has limitations and can result in variable degrees of failure^3^^,^ 26 ^,^ 27. Among several options to ameliorate the treatment, one promising procedure could be the HBO.

The use of HBO has proved to be a relevant topic to be investigated due to its ability to protect against tissue ischemia. Moreover, the use of random skin flaps in rodents as an experimental animal model could contribute to a better understanding of the role of HBO against tissue ischemia.

The increase in tissue oxygen tension caused by HBO remains for more than 2 h after the end of the HBO session. Such phenomenon is called on-off effect. According to the principle’s physics of gases diffusion (laws of Boyle and Henri)^24^, HBO provides a favorable mechanism for the tissue when compared to normobaric pressures.

The protective mechanism of HBO noted in ischemic tissues assumes that there are multiple protection routes attributed to the increase in plasma oxygen, caused by the increase in ambient pressure. These phenomena increase tissue tolerance to ischemia due to reduction in the inflammatory process^2^^,^ 7 ^,^ 23 ^,^ 24 ^,^ 27 ^,^ 28. It has also been reported that HBO promotes increased blood flow in the microcirculation (angiogenesis) and reduced platelet aggregation. These associated characteristics are combined with a better ability of the plasma to carry dissolved oxygen^29^^,^ 30.

In a previous study, an increase in the viability of the random flap was noticed attributed to the use of HBO for seven days after the operation. This positive effect was probably due to the maintenance of tissue oxygen pressure in poorly perfused and ischemic tissue areas, until the blood flow in the flap increases due to the response of the tissue to angiogenesis^1^^,^ 25. It should be mentioned that angiogenesis is modulated by growth factors that are located in endothelial cells and extracellular matrix^30^^,^ 31.

VEGF-A is associated with the beginning of the process of vascular neoformation and is considered a reliable monitor of the process^31^. VEGF-A is also an angiogenesis and/or neoangiogenesis monitor used to control various pathological situations, including chronic ischemia of arterial origin in skeletal and cardiac muscles^33^^,^ 34, as well as in vascularization studies of neoplastic tissues^32^^,^ 35.

Immunohistochemistry for VEGF-A detection is a widely used technique to identify endothelial cells in blood vessels, which allows the quantification of blood vessels present in a certain area of the tissue field seen under microscopy. Thus, in this study, it was chosen to quantify the number of blood vessels immunopositive to VEGF-A (per mm^2^) to assess a possible effect of HBO to promote angioneogenesis in a skin flap model *in vivo*, a method already mentioned in other publications^36^. The protocol applied in this research corresponds to that one used in the practice of HBO, and, despite the criteria used, a promising response was identified with the standardization of the application of HBO in this format.

Descriptive microscopy data showed no differences in VEGF-A expression between GE and GE/HBO groups, with a similar number of blood vessels in the three regions studied. This data indicated that there was no effect regarding VEGF-A expression on tissue not submitted to the surgical procedure. The amount of blood vessels immunostained to VEGF-A was lower in animals that underwent only the surgical procedure, when compared to groups subjected to HBO (Table 1).

The qualitative description evidenced the action of HBO when compared to the quantification of blood vessels (Fig. 2 and Table 1), and the result indicated that the use of HBO promotes tissue viability in the experimental condition applied. It is a consensus in the literature that the increase in oxygen tension resulting from HBO, when blood pressure becomes elevated to 1,000-1,500 mmHg due to dissolution in plasma, improves cell function and promotes angioneogenesis^4^.

In a previous report similar to the one studied in the present article, the authors described that the model used with interposition of the polyethylene film may be responsible for the lack of VEGF expression in the treated groups. It was postulated that the stimulation for angiogenesis is dependent on the bed on which flap was replaced after it was made. If there were no contact with the bed, the growth of new vessels may have been delayed^37^.

According to the results of GER and GER/HBO groups, it is highlighted the importance of stimulating the contact of the flap with the bed on which it was replaced for angiogenesis, with a significant response in the group submitted to HBO. However, further studies need to be carried out in order to investigate the use of HBO in compromised skin flaps^4^^,^ 38.

This research showed that there is a favorable effect of HBO on vascularization and improved viability of random ischemic flaps in rats. However, it should be mentioned that the evaluation of a single biomarker does not really prove the effect of hyperbaric therapy on flap viability. Thus, new biomarkers must be further investigated. Moreover, the results described here were obtained in animals within the proposed model, which does not allow to extend the use of HBO in the injury of other tissues. Despite this limitation, these results open research perspectives in the HBO field, to elucidate the effect of HBO in tissue ischemia.

## Conclusion

HBO is associated with an increase in blood vessel growth and viability in random skin flaps of rats, thus paving the way to further studies to confirm and clarify the mechanism of action.
